# Psychostimulants As Cognitive Enhancers in Adolescents: More Risk than Reward?

**DOI:** 10.3389/fpubh.2017.00260

**Published:** 2017-09-26

**Authors:** Kimberly R. Urban, Wen-Jun Gao

**Affiliations:** ^1^Department of General Anesthesia, Division of Stress Neurobiology, Children’s Hospital of Philadelphia, Philadelphia, PA, United States; ^2^Department of Neurobiology and Anatomy, Drexel University College of Medicine, Philadelphia, PA, United States

**Keywords:** methylphenidate, psychostimulant, adolescence, young adult, learning drugs, drug abuse, psychiatry

## Abstract

Methylphenidate and other psychostimulants, originally developed to treat attention deficit-hyperactivity disorder, are increasingly abused by healthy adolescents and adults seeking an advantage in scholastic performance and work productivity. However, how these drugs may affect cognitive performance, especially in the young brain, remains unclear. Here, we review recent literature and emphasize the risks of abuse of psychostimulants in healthy adolescents and young adults. We conclude that while the desire for cognitive enhancement, particularly with rising costs of education and increasingly competitive nature of scholarship programs, is unlikely to diminish in the near future, it is crucial for the scientific community to thoroughly examine the efficacy and safety of these stimulants in healthy populations across development. The current dearth of knowledge on the dose–response curve, metabolism, and cognitive outcomes in adolescents following methylphenidate or other psychostimulant exposure may be perpetuating a perception of these drugs as “safe” when that might not be true for developing brains.

Attention deficit-hyperactivity disorder (ADHD) is one of the most commonly diagnosed childhood psychiatric disorders, affecting 5% (1) to 11% (2) of children aged 4–17 years old. Diagnosis of ADHD has increased in the United States during the past two decades (3, 4). The exact cause of this increase is unknown, but may be attributable to better diagnostic tests, increasing awareness of ADHD, or even perhaps conflicts of interest (doctors appeasing concerned parents). Diagnosis is largely subjective, and stringency of adherence to the DSM-V criteria varies: many children are taken to a primary-care physician on the advice of a teacher, and given methylphenidate (MPH) or amphetamine (AMPH) as a first-line treatment, often without rigorous psychiatric testing (5). ADHD is thought to arise from a deficit of the neurotransmitters dopamine (DA) and norepinephrine (NE) in the prefrontal cortex (PFC), which leads to impairments in executive function, causing symptoms of impulsivity, locomotor hyperactivity, and impairments in judgment and social behavior, and can lead to devastating impairments in scholastic and job performance and deteriorating social relationships if left untreated (6, 7). Functional magnetic resonance imaging (fMRI) and positron emission tomography (PET) imaging taken from individuals diagnosed with ADHD have shown reductions in blood flow in the PFC (8, 9). Proper function of PFC relies on the concentrations of the neurotransmitters DA and NE; these neurotransmitters exert control over executive function in an inverted-U curve manner: both insufficient and excessive levels result in impairments in PFC functions (10, 11). The PFC undergoes extensive synaptic pruning throughout puberty and into early adulthood. This delayed maturation may impart particular vulnerability of the adolescent brain to perturbations such as drug abuse, injury, and stress (12). In fact, many psychiatric illnesses, including ADHD, that manifest with impairments in executive function, are largely diagnosed and treated in juveniles and adolescents (13). ADHD and stress-related psychiatric disorders include impairments in working memory as a common symptom, further implicating PFC impairment in their pathology.

Psychostimulants are currently the first-line treatment for ADHD in both children and adults; of these, MPH is the most widely prescribed (14, 15). MPH exerts its therapeutic effect by blocking the function of the DA transporter (DAT) and norepinephrine transporter [NET, thereby increasing the bioavailability of the neurotransmitters and correcting the deficit thought to cause ADHD (16–18)]. The first stimulant approved for ADHD treatment AMPH (Adderall©) blocks reuptake but also increases vesicular release of DA; the effect on DA release is the main action at low doses (19). There is a large body of research supporting the conclusion that psychostimulant treatment reduces symptoms of ADHD, particularly hyperactivity (20). However, how other psychostimulants may affect cognitive performance is less clear, due to varying dosages, varying ages of subjects, and the fact that many tests of executive function contain non-executive domains on which improvement is noted after psychostimulant treatment. For example, MPH is effective at improving performance on a simple reaction time, task-switching paradigm, focused attention, word-matching, and go/no-go tasks in children with diagnosis of ADHD, but not spatial working memory (SWM), pattern recognition, or divided attention tasks (21–30). Regardless, stimulant medications seem to improve cognitive function in an inverted-U curve manner, with lower doses improving and higher doses impairing various aspects of cognition (17).

Animal studies have been used to examine safety and efficacy of MPH and similar psychostimulants such as AMPH for decades; rats possess the same neurotransmitter systems and pathways as humans and have a more primitive, but similar, PFC. Studies of stimulant actions in rodents have shown varied, often contradictory effects, due to inconsistencies in dosing. For example, MPH caused hyperlocomotion and chasing in a paired open-field task, reduce learning retention, alter gene expression, and produce stereotypies; however, these studies used high doses of MPH (31–37). Low-dose MPH has been shown to improve performance on sustained attention, signal detection, and attentional set-shifting (38–40). For a more thorough historical review of rodent MPH studies, see Ref. (41). Adult rodent studies and studies on adult human volunteers suggested that psychostimulant improvement of cognition was not “paradoxical effect” observable only in ADHD and models of ADHD, but also presented in healthy individuals. Further recent studies corroborated the theory that low-dose psychostimulant treatment (doses that correspond to those given to ADHD patients) appears to enhance prefrontal cortical-dependent functions and cognitive performance in healthy individuals in a similar manner to ADHD patients (42–45). This led to consideration of MPH as a nootropic, or cognitive-enhancing, drug.

Today, MPH is increasingly abused by adolescents and adults seeking an advantage in scholastic performance and work productivity. It is used to aid memory when studying for exams and to improve focus and wakefulness (46, 47). MPH and other similar substances are also highly abused by members of the military to improve attention in high-stress situations and combat the effects of sleep deprivation (48). Prevalence reports range from 2 to 20% of respondents admitting to cognitive enhancement (47, 49–51). There is extreme controversy regarding cognitive enhancement, with physicians and the public questioning safety and morality of the artificial augmentation of cognition (52–55) and even doubts about the true prevalence of misuse (56). Despite the rising abuse of MPH among adolescents and young adults, the basis for its safety and efficacy as a nootropic arises from studies performed on adult rodents and human volunteers; there is little information about the potential adverse behavioral and cognitive effects of stimulant treatment in normal adolescents. Recent precious little research has been conducted using adolescent or juvenile rodents until the last 5 years. These studies have revealed strikingly different effects than adult rodent studies. For example, adolescent MPH exposure was found to reduce social play, impair pattern learning and reversal learning, increase locomotor hyperactivity, and response to cocaine, sometimes lasting into adulthood (57–60). Early exposure to MPH has also been shown to result in increased anxiety lasting into adulthood and alter circadian rhythms (61–65). However, many of the recent studies on adolescent rats have not been consistent in their dosing regimens, leading to concern as to the therapeutic relevance of the results. A therapeutically relevant dose range that results in peak blood plasma levels equivalent to those measured in successfully treated patients (8–40 ng/dL) has been established for adult rats at 0.5–1 mg/kg injected intraperitoneally, but the dose–response range has not been systematically examined for adolescent rats (18). We recently reported that a single dose of 1 mg/kg intraperitoneal given at 17–25 postnatal days in the rat resulted in significant depression of neuronal activity and synaptic transmission in the layer V pyramidal neurons of the PFC. This same dose resulted in the expected increase of activity in those same neurons in adult rats (66). These results suggest that there is an age-dependent effect of MPH in the PFC, and that the juvenile brain may be hypersensitive to the effects of psychostimulants, and even a low dose may push the healthy developing brain into a hyperdopaminergic and hyperadrenergic state. We further examined the effects of a single 1 mg/kg dose of MPH on glutamate receptors and plasticity in the juvenile rat, and reported that MPH selectively reduced levels of NR2B-containing NMDA receptors, and abolished short-term facilitation while enhancing long-term potentiation (LTP) and decreasing long-term depression (LTD) (67). Excessive DA levels can lead to reduced expression of NR2B-containing NMDA receptors *via* activation of the glycogen synthase kinase (GSK-3β) pathway, disrupting β-catenin association with NR2B and allowing ubiquitination (68).

Despite its widespread misuse and ready availability, MPH is not the only psychostimulant or catecholaminergic agent considered for its utility as a cognitive enhancer. Amphetamine has been shown to improve consolidation and recall. However, it is associated with a variety of neurotoxic negative effects, including synaptic terminal degradation and neuronal chromatolysis in cortex and striatum, and permanent loss of DA uptake sites in striatum and nucleus accumbens (69–74). Furthermore, AMPH can induce a schizophrenia-like psychosis marked by hallucinations, paranoia, panic, and hyperactivity (75–77). AMPH produces more rapid sensitization than MPH and induces a robust subjective “high” through its actions on serotonin, making it more addictive than MPH and less popularly used as a nootropic (78). In the PFC, both NE and DA are taken up by the NET due to limited expression of the DAT (79) and the relatively high affinity of DA for NET as compared with DAT (80, 81). Therefore, the transporter selectivity of a compound may influence its utility as a cognitive enhancer and its abuse potential. AHN 2-005, a DAT-selective compound, significantly increased extracellular levels of both NE and DA in the PFC at a cognition-enhancing dose that lacked locomotor activating effects, similar to MPH (82). However, AHN 2-005 produced a larger increase in extracellular DA in the nucleus accumbens than PFC. Although MPH selectively affected DA and NE levels in PFC (18, 82), it has yet to be considered a cognitive enhancer, largely due to its unavailability in the market.

Another important ADHD medication is atomoxetine (Strattera©), which is a non-stimulant and is also rarely considered a cognitive enhancer despite its nootropic and abuse potential. While there are far fewer peer-reviewed studies of atomoxetine as a cognitive enhancer, Internet searches reveal multiple forums and discussions of its efficacy and safety among individuals taking this drug illicitly. Atomoxetine selectively inhibits NET, with no appreciable actions on the DAT or other systems associated with abuse potential (83–85). Therefore, atomoxetine lacks the central nervous system stimulant effects of MPH and AMPH, reducing the risk of cardiovascular events (86). However, atomoxetine has been shown to bind to serotonin transporters, which may blunt its nootropic effects, as extrasynaptic serotonin can impair episodic memory and recall and may be anxiogenic (87). See Table 1 for a summary of relevant psychostimulants and ADHD medications considered for cognitive enhancing usage in healthy adolescents and young adults.

**Table 1:** Summary of psychostimulants and compounds affecting catecholamine reuptake considered for cognitive enhancement abilities.

Chemical	Brand name(s)	Mechanism of action	Neurotransmitters systems affected	Abuse potential
Methylphenidate	Concerta© and Ritalin©	NET and DAT inhibitor	Norepinephrine and dopamine	Moderate. Does not produce addiction, but is commonly sold off-label and taken by adolescents and young adults for nootropic effects
Amphetamine	Adderall© and Adzenys XR-ODT	NET, DAT, and SET inhibitor	Norepinephrine, dopamine, and serotonin	High. Addictive, produces subjective “high.” Commonly abused and readily available
Atomoxetine	Strattera©	NET inhibitor	Norepinephrine (potentially serotonin)	Low. Does not produce addiction, no stimulant actions, but is readily available
AHN 2-005	N/A	DAT inhibitor	Dopamine	Negligible. Not commercially available

*DAT, dopamine transporter; NET, norepinephrine transporter; SET, serotonin transporter*.

Despite these studies, it is still unclear how NET and DAT individually influence catecholamine balance in PFC; however, at least some evidence suggests that both are important (88). Furthermore, how low doses of psychostimulants preferentially target catecholamines in PFC without affecting other brain regions is unknown, and it may be unfeasible to elucidate potential mechanisms *in vivo* due to the common ligands and overlapping functions of the NET and DAT, as well as the fact that most compounds have at least some reactivity with both. How can NET control reuptake of both catecholamines in PFC if it is dominant to DAT? Furthermore, it is important to elucidate the contributions of DAT and NET to the uptake of NE and DA across development. Specifically, is PFC DAT expression already low compared to NET at birth, or does the expression of DAT progressively decrease during development? Since MPH induces opposite effects on neuronal activity, synaptic transmission, and plasticity in the juvenile versus adult animal PFC in a clinically relevant dose for adult (66, 89), it is important to explore the developmental expression and function of NET and DAT in the PFC.

What do our results and the results of similar studies mean for the adolescent taking MPH or similar psychostimulants? How does this differ from the consequences for an adult taking psychostimulants? MPH and other psychostimulants are thought to exert their therapeutic effect by raising levels of DA and NE specifically in the PFC; this leads to increases in neuronal activity and enhanced prefrontal cortical top–down control of executive functions (17). However, DA and NE exert an “inverted-U” curve of effects in the PFC: too little and the PFC is not able to function, resulting in impulsiveness, inattentiveness, locomotor hyperactivity, and poor decision-making, too much and the signal-to-noise ratio of neuronal firing is lost, resulting in scattered attention, stereotypic movements, hyperactivity, and impulsiveness (10, 90). In individuals with ADHD, and low levels of DA and NE, psychostimulants would raise the levels into the optimal range, and in healthy adults, it appears that low-dose psychostimulants may further optimize catecholamine levels and provide improvements in executive function (91–98). Thus, psychostimulants given at low doses similar to those used to treat ADHD may indeed provide an effective and largely safe cognitive enhancement, as the PFC of adults has finished maturing (11, 12, 99).

However, in the adolescent brain, levels of DA and NE are naturally higher, as the PFC development is ongoing and synaptic pruning has not been completed; thus, adding psychostimulants likely pushes the levels of DA and NE beyond the optimal range and into excessive levels (12). This is consistent with impairments in pattern learning and object-memory, reduced pyramidal neuron activity, and reduced NR2B-containing NMDA receptor levels seen in our studies (66, 67). The precise function of LTP in the PFC has not been elucidated; however, it has been hypothesized that if persistent firing and short-term facilitation are a measure of working memory, then LTP might be a neuronal correlate of sustained attention and memory consolidation/learning. If this is the case, we can predict that psychostimulant abuse by adolescents and juveniles could result in impairments in working memory and behavioral flexibility, but enhanced sustained attention. Thus, in a scholastic setting, adolescents taking MPH off-label or in abusing the drug may appear to be improving; however, rigid testing of working memory and cognitive flexibility might reveal impairments that could negatively impact their lives. For example, the ability to quickly redirect attention is critical for sports and driving: on the road, one must be able to notice approaching cars and quickly determine the optimal response, shifting gaze from the road to one’s dashboard, and back again. In a work setting, especially in jobs that require management of subordinates or teamwork, the roles of individuals may change, and one must be able to evaluate quickly one’s performance. Inability to approach problems from different paths could lead to poor performance at work, leading to reduced pay or termination of employment. Finally, behavioral rigidity could potentially raise the risk of drug addiction, as inability to terminate behaviors associated with the taking of drugs is a common obstacle to recovery and impaired flexibility has been shown in multiple addiction phenotypes in humans (100–102).

While it is currently unclear if the impacts of low-dose MPH on the juvenile PFC are permanent, our research suggests that recovery depends on dose; neuronal activity and synaptic transmission recovered to control levels within 1 week following 1 mg/kg but did not recover even 10 weeks after 3 or 9 mg/kg treatment in the rat (66). Individuals abusing stimulants on college and high-school campuses are often exposing themselves to much higher doses than are typically clinically prescribed, and doing so without the benefit of building tolerance; thus, they may be particularly vulnerable to long-lasting alterations in prefrontal cortical function. The desire for cognitive enhancement, particularly with rising costs of education and increasingly competitive nature of scholarship programs, is unlikely to diminish in the near future; therefore, it is crucial for the scientific community to thoroughly examine the efficacy and safety of each candidate substance. The current dearth of knowledge on the dose–response curve, metabolism, and cognitive outcomes in juveniles and adolescents following MPH or other psychostimulant exposure may be perpetuating a perception of these drugs as “safe” for any age when that might not be true. Until the research is completed to give us a more thorough understanding of the drugs’ actions in the developing PFC, off-label use of psychostimulants and nootropics may present more risk than reward for adolescents.

## Author Contributions

All authors listed have made a substantial, direct, and intellectual contribution to the work and approved it for publication.

## Conflict of Interest Statement

The authors declare that the research was conducted in the absence of any commercial or financial relationships that could be construed as a potential conflict of interest.
